# CRISPR/Cas9 technology: applications in oocytes and early embryos

**DOI:** 10.1186/s12967-023-04610-9

**Published:** 2023-10-24

**Authors:** Yi-ran Zhang, Tai-lang Yin, Li-quan Zhou

**Affiliations:** 1 Institute of Reproductive Health, Tongji Medical College, Huazhong University of Science and Technology, Wuhan, Hubei China; 2 Reproductive Medical Center, Renmin Hospital of Wuhan University & Hubei Clinic Research Center for Assisted Reproductive Technology and Embryonic Development, Wuhan, China

**Keywords:** CRISPR, Oocyte, Embryo, Development, Human germline genome editing

## Abstract

CRISPR/Cas9, a highly versatile genome-editing tool, has garnered significant attention in recent years. Despite the unique characteristics of oocytes and early embryos compared to other cell types, this technology has been increasing used in mammalian reproduction. In this comprehensive review, we elucidate the fundamental principles of CRISPR/Cas9-related methodologies and explore their wide-ranging applications in deciphering molecular intricacies during oocyte and early embryo development as well as in addressing associated diseases. However, it is imperative to acknowledge the limitations inherent to these technologies, including the potential for off-target effects, as well as the ethical concerns surrounding the manipulation of human embryos. Thus, a judicious and thoughtful approach is warranted. Regardless of these challenges, CRISPR/Cas9 technology undeniably represents a formidable tool for genome and epigenome manipulation within oocytes and early embryos. Continuous refinements in this field are poised to fortify its future prospects and applications.

**Supplementary Information:**

The online version contains supplementary material available at 10.1186/s12967-023-04610-9.

## Introduction

**Fig. 1:**
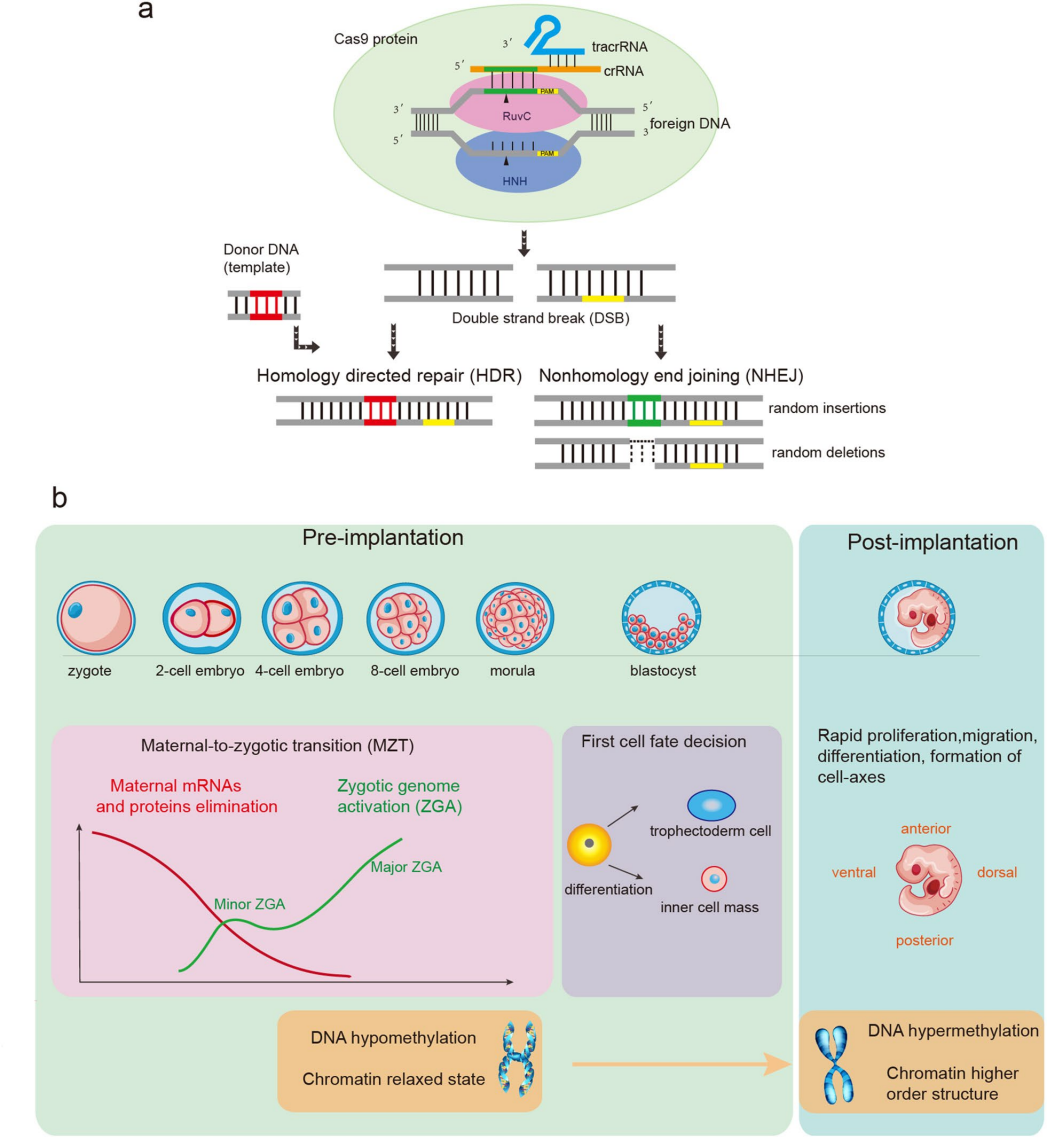
Schematic of CRISPR system and early embryo development. **a** Schematic of the CRISPR system structure and gene repair mechanism. The crRNA (blue) and tracrRNA (orange) direct the Cas9 protein to target the gene of interest, and two nuclease domains (HNH and RuvC) cut the corresponding gene sites to generate DSB. HDR and NHEJ mechanisms repair DNA sequences, and the different outcome is shown in the diagram. **b** Schematic of early embryo development and a series of epigenetic events, including maternal RNA/protein elimination (red curve), zygotic genome activation (ZGA, green curve), and the changes of DNA methylation and chromatin structures, are also shown

The precise editing of mammalian genome lays the foundation for genetic studies and investigations on germ cells [1]. Clustered regularly interspaced short palindromic repeats (CRISPR), uncovered from the immune system of bacterial and archaea in response to foreign phage invasion [2], is a highly efficient DNA editing tool (Fig. 1a) [3–5]. This mechanism relies on the help of the small guide RNA (sgRNA), which combines crRNA and tracrRNA for targeting genes, as well as the Cas protein for cleavage [6–8]. The dsDNA of 3 bp upstream of the protospacer adjacent motif (PAM) is cleaved by HNH and RuvC nuclease domains [3, 6, 7], generating a site-specific blunt-ended double-strand break (DSB) [6, 9, 10]. Moreover, both HNH and RuvC nuclease domains can be inactivated through mutagenesis [3], causing the development of nuclease dead Cas9 (dCas9) that can be fused to transcriptional activators or repressors for regulation of the target gene expression [11]. DSBs can be repaired via homology-directed repair (HDR), non-homologous end joining (NHEJ), or microhomology-mediated end joining (MMEJ) processes, but the repaired DSB may harbor random insertions and/or deletions at the cleavage site [12–14]. Notably, this technology performs well in the manipulation of the mammalian germline genome despite the challenges and ethical considerations it has faced [1].

Several critical biological events accompany the early embryonic development process (Fig. 1b) [15–17]. Fertilization of oocyte by sperm causes the maternal-to-zygotic transition (MZT), encompassing maternal mRNA elimination and zygotic genome activation (ZGA) [15, 17–19]. Multiple epigenetic modifications take place in early embryos and are critical in pre-implantation development [20–23]. DNA methylation and dynamic histone modifications such as trimethylation of histone 3 lysine 4 (H3K4me3) and histone 3 lysine 27 acetylation (H3K27ac) often arise across diverse genomic regions and are essential for specific developmental processes through transcriptional regulation [24–28]. Notably, the chromatin structure of embryos during cleavage stages is in a uniquely relaxed state featuring totipotency, and the single-cell chromatin overall omic-scale landscape sequencing (scCOOL-seq) technique demonstrated that the most dramatic chromosome reprogramming events in mammals took place in cleavage-stage embryos [18, 29–33]. However, many of the linked studies are descriptive, and regulatory networks, as well as related mechanisms remain largely undefined.

As an efficient and inexpensive genome editing tool, the CRISPR/Cas9 system has received increased attention in recent years. In this review, we primarily focus on the current use of CRISPR/Cas9 in oocytes or early embryos, as well as the limitation of this technology. With the assistance of this technology, it is possible to fix gene mutations, modify gene expression levels by epigenetic manipulation, and visualize proteins directly, to answer important scientific questions and treat diseases in human and other species [34–40]. However, it is a considerable challenge to overcome the limitations of the CRISPR/Cas9 technique, including on-target effects, off-target effects, and HDR rate [41–44] (Additional file 1: Table S1). While CRISPR systems are gradually improved, previously unrecognized errors in gene editing are continuously revealed [45, 46], and genome editing oversights may introduce uncertainty into the health of offspring. These limitations and linked ethical issues should be addressed before this technology is applied further in mammalian oocytes and embryos.

## Application of CRISPR in mammalian cells

### Gene editing

**Fig. 2:**
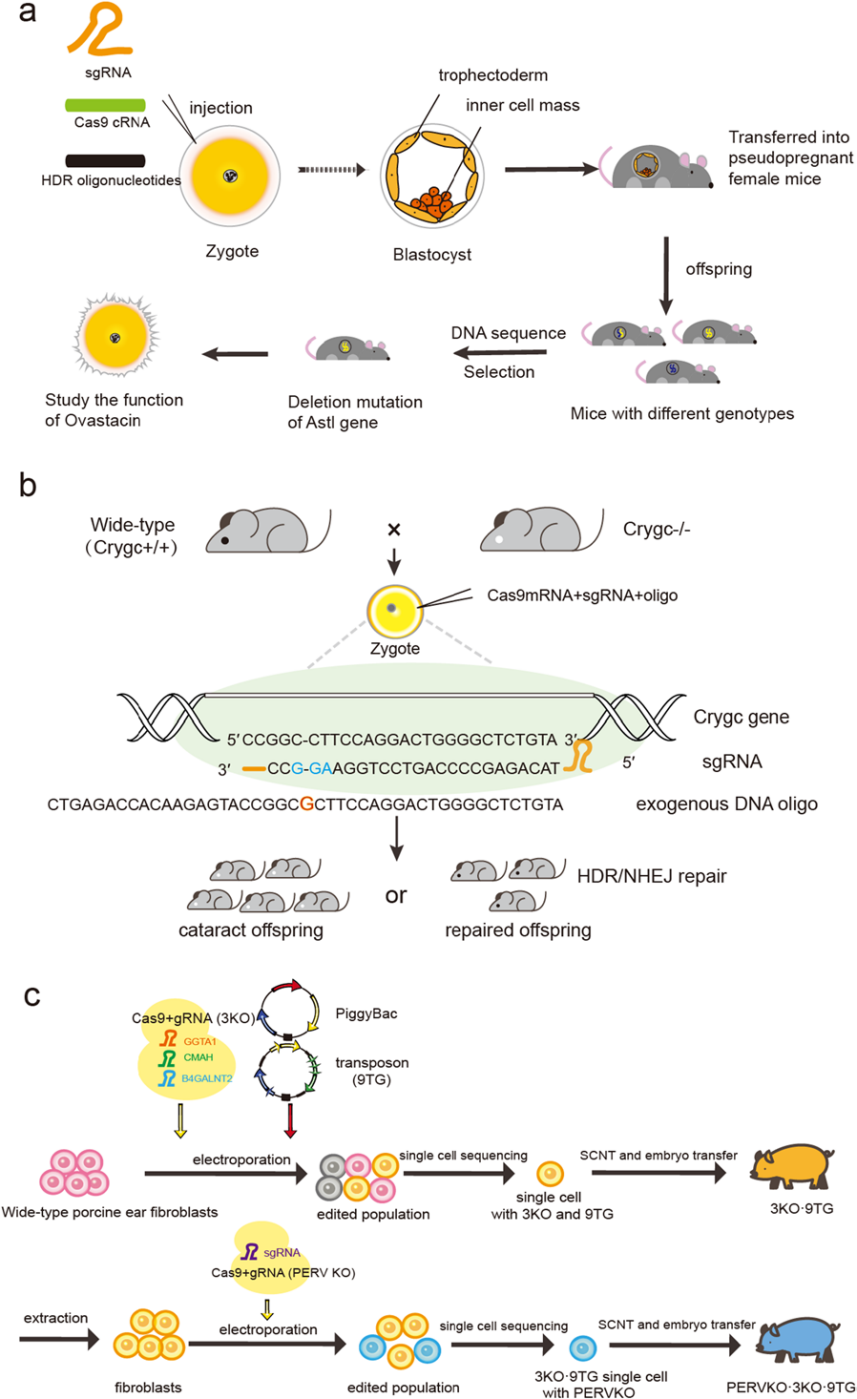
Diagram of genome editing in mammals. **a** Diagram summarizing the processes for gene editing of *Astl* in mice using the CRISPR/Cas9 system, including microinjection, embryo transfer, identification of founders, obtaining homozygous mutants through breeding, and performing phenotype examination and mechanism investigation. **b** Diagram summarizing the correction progression of a genetic defect in a cataract mouse model. The zygotes from wild-type mice crossed with Crygc^−/−^ mice were used. Some offspring embryos showed normal phenotypes following microinjection of Cas9 mRNA, sgRNA and exogenous DNA oligos into heterogeneous zygotes. **c** The processes to generate PERVKO·3KO·9TG pigs. In the first round of engineering, 3KO·9TG pigs were obtained by SCNT using CRISPR/Cas9-edited porcine fibroblasts. In the next round of engineering, the candidate fibroblasts from 3KO·9TG pigs were isolated and further edited by the CRISPR/Cas9 system, and PERVKO·3KO·9TG pigs were obtained using SCNT.

CRISPR/Cas9 has been used to generate DNA mutations to produce homozygous loss-of-function animals. For example, the specific domain within the *Astl* gene encoding Ovastacin was removed using CRISPR to identify the role of Ovastacin in preventing sperm binding at zona pellucida following fertilization [47, 48]. In general, the CRISPR system, including sgRNA and HDR oligonucleotide, was introduced into mouse zygotes to remove the seven amino acids of the *Ovastacin* gene using microinjection (Fig. 2a) [48, 49]. Loss of *Crygc* gene resulted in cataracts in mice, and it has been reported that *Crygc* gene deletion could be fixed using the CRISPR system in mice (Fig. 2b) [50]. The founders were able to encode the *Crygc* gene and transmit it to offspring successfully [50]. Moreover, genetic correction of Duchenne muscular dystrophy (DMD) mutations by using CRISPR/Cas9-mediated techniques was recently reported [51–54]. For instance, CRISPR in mouse zygotes was validated to correct DMD mutations in a mouse model [52].

Moreover, CRISPR has also been introduced to modify porcine genes and has been instrumental in developing xenotransplantation. Encouragingly, it has been used to inactivate porcine endogenous retroviruses (PERVs) by repressing PERV reverse transcriptase (pol) gene and to enhance compatibility with the human immune system by editing immune-related genes in pigs (Fig. 2c). Somatic genes were successfully manipulated using CRISPR/Cas9 to generate heritable pigs via somatic cell nuclear transfer (SCNT) [55]. Notably, the researchers combined CRISPR and transposon technologies to modify 13 genes in the large animal model, demonstrating the engineering power of CRISPR/Cas9 in the mammalian germline [55].

Recently, maternal mutant embryos were acquired rapidly through microinjection of multiple sgRNAs and other CRISPR components to eliminate corresponding maternal products in zebrafish [56]. Crispants, a new technology derived from CRISPR/Cas9, is helpful for investigating maternal-effect genes [57]. To date, crispant has been shown to be successful in identifying maternal-effect genes such as *kpna7* in zebrafish by inducing high-frequency biallelic editing of the germ line [57].

Mitochondria, as the energy-producing organelles of eukaryotic cells, contain unique mitochondrial DNA (mtDNA), which contributes to maternally inherited genetic disorders. The mitochondrially targeted engineered nucleases such as mitochondrially targeted TALENs (mitoTALENs) and mitochondrially targeted zinc finger nucleases (mtZFNs) have been reported to limit pathogenic mtDNA mutations in mouse oocytes and improve the quality of aged eggs [58, 59]. Using the CRISPR/Cas9 system, it is gradually applied in treating mitochondrial diseases. Jo et al. reported the manipulation of mtDNA using the CRISPR/Cas9 system through the creation of a mitochondria-targeted Cas9 (mitoCas9) localized to mitochondria together with the specific gRNA to cleave mtDNA without affecting genomic DNA [60].

Collectively, the CRISPR/Cas9 system has been successfully applied in mammalian oocytes and embryos across various animal models including mice and pigs, and previously confronted difficulties in the treatment of some diseases may be addressed by directly altering the genomic sequences. Moreover, the application of genome editing techniques in mammalian oocytes and embryos will significantly enable the mechanistic study of developmental events at these stages, and a complete understanding of molecular mechanisms will provide valuable information for technical advancement.

### Transcriptional regulation

**Fig. 3:**
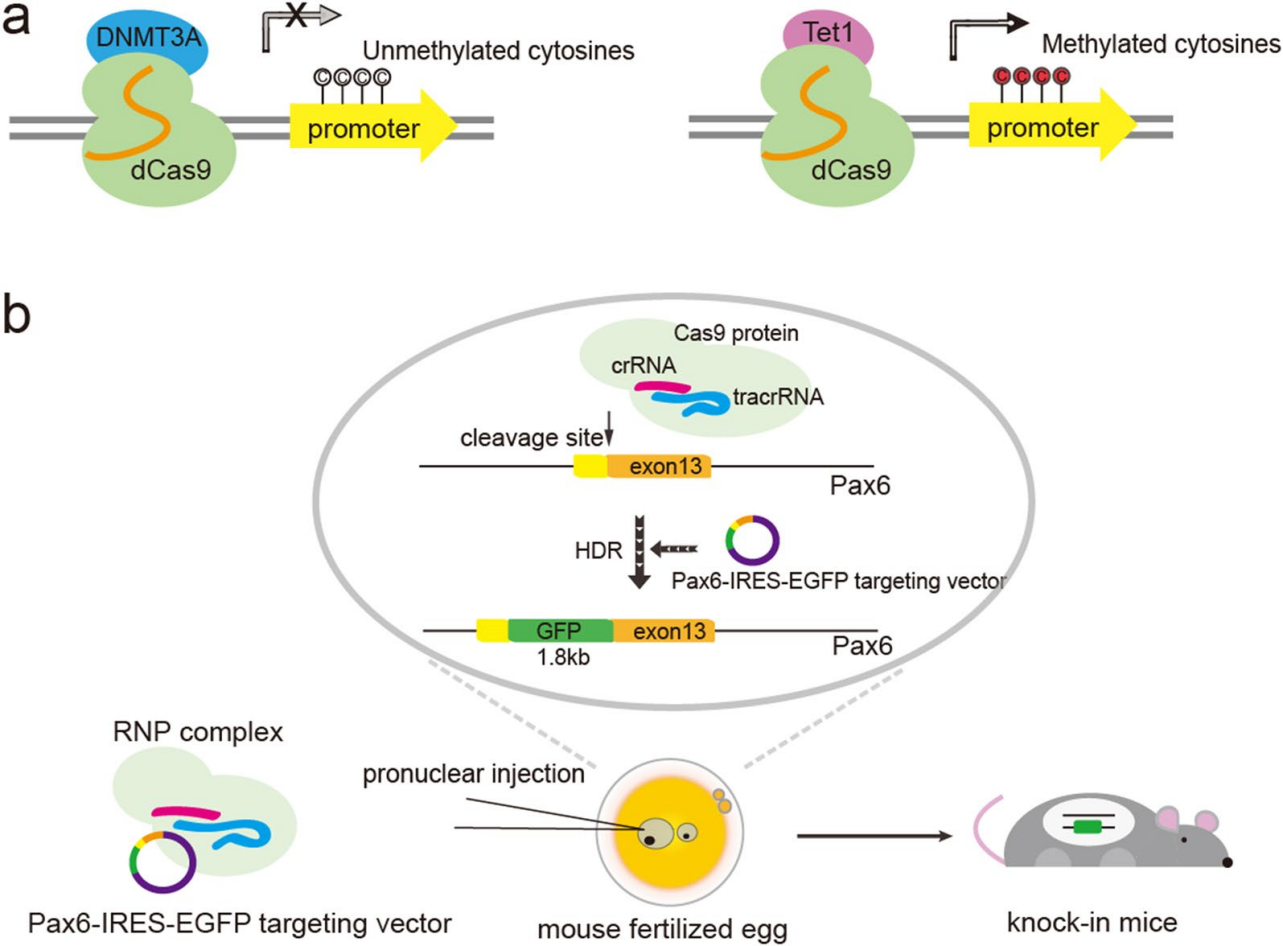
Diagram of transcriptional regulation and protein labeling by CRISPR/Cas9 system. **a** Diagram showing that the inactive Cas9 protein can be fused to epigenetic modifiers like DNMT3A and TET1 to alter gene expression by modifying the methylation state of cytosine in the specific promoter. **b** Diagram of pronuclear injection process to generate reporter mice. The RNP complex which contained Cas9 protein, crRNA and tracrRNA, and Pax6-IR4ES-EGFP targeting vector was injected into mouse fertilized eggs to obtain knock-in mice

CRISPR is a flexible tool, particularly catalytically inactivated dCas9, which has been developed as a DNA-targeting module for epigenome engineering [9, 36, 61, 62]. With the successful application of novel gene editing tools such as dCas9-v64 in the early stage, the fusion of dCas9 to various transcription regulators or modifying enzymes has gradually been shown to be effective in regulating gene expression, including P300, VPR, KRAB, MECP2, TET, and DNMT [37, 62–66]. For example, studies have shown that the fusion of dCas9 with DNMT3a or TET1 allows for the silencing or activating of endogenous reporters respectively, by targeting promoter sequences [61] (Fig. 3a). The application of dCas9-DNMT genome editing has been demonstrated to reduce PLPP3 expression by increasing 5-methylcytosine (5mC) [67]. There is also ample literature demonstrating the effectiveness of this type of editing [39, 67–69]. Individual mammalian oocytes and embryos can be used to edit the methylation status of target genomic regions through microinjection of specific methylation editing systems, such as the dCas9-TET/DNMT complex, which can correct familial Angelman syndrome in a mouse model [70]. Additionally, an improved CRISPR system, which includes sgRNA and dCas9-*Dnmt3a*, was applied to edit seven genomic imprinting regions simultaneously in single unfertilized oocytes, and these oocytes produced offspring successfully following fertilization [38]. The investigation of oocyte methylation has been promoted, bringing a unique strategy to inhibit or correct maternally transmitted nongenetic diseases or disorders.

Moreover, alterations in chromatin looping can also modulate gene expression [61]. Topologically associating domains and gene loops governed by architecture proteins like CTCF (CCCTC-binding factor) and cohesion complex are important chromatin elements [71]. When DNA methylation of CpGs was introduced via the targeting of dCas9-DNMT3a to two CTCF binding sites, the interaction between enhancers and nearby gene loops was identified to be increased, facilitating gene activation [61].

Collectively, the CRISPR/Cas9 system can tune gene expression at the transcriptional level through epigenetic editing of chromatin modifications without altering genomic sequences.

### Protein-targeted regulation and visualization

Endogenous target proteins may be degraded through the CRISPR/Cas9-introduced auxin-inducible degradation (AID) system. For instance, degradation of maternal proteins may be efficiently induced using the AID system in the ovary and early embryo of *Drosophila* [72].

CRISPR/Cas9 technology enables systematic studies of protein localization and protein-protein interactions with the assistance of tag-based proteomics [73–75]. The Pax6-IRES-EGFP knock-in mouse lines have been established to express endogenous EGFP in the *Pax6* locus, and the visualization of endogenous PAX6 dynamics was obtained using an optimized CRISPR/Cas9-mediated technique (Fig. 3b) [76, 77]. Additionally, the complex system containing EGFP-tagged dCas9 and site-specific sgRNAs allows for the visualization of repetitive element-containing chromatin regions such as telomeres, centromeres, and satellite DNAs in the genome [40]. In this way, CRISPR imaging was successfully employed to assess the dynamics of telomeres during telomere elongation and the dynamic behaviors of the MUC4 loci during mitosis [40].

Overall, the CRIPSR/Cas9 system can label endogenous proteins to tailor their stabilities and demonstrate their characteristics, including localization and dynamics.

## Application of CRISPR in human cells

**Fig. 4:**
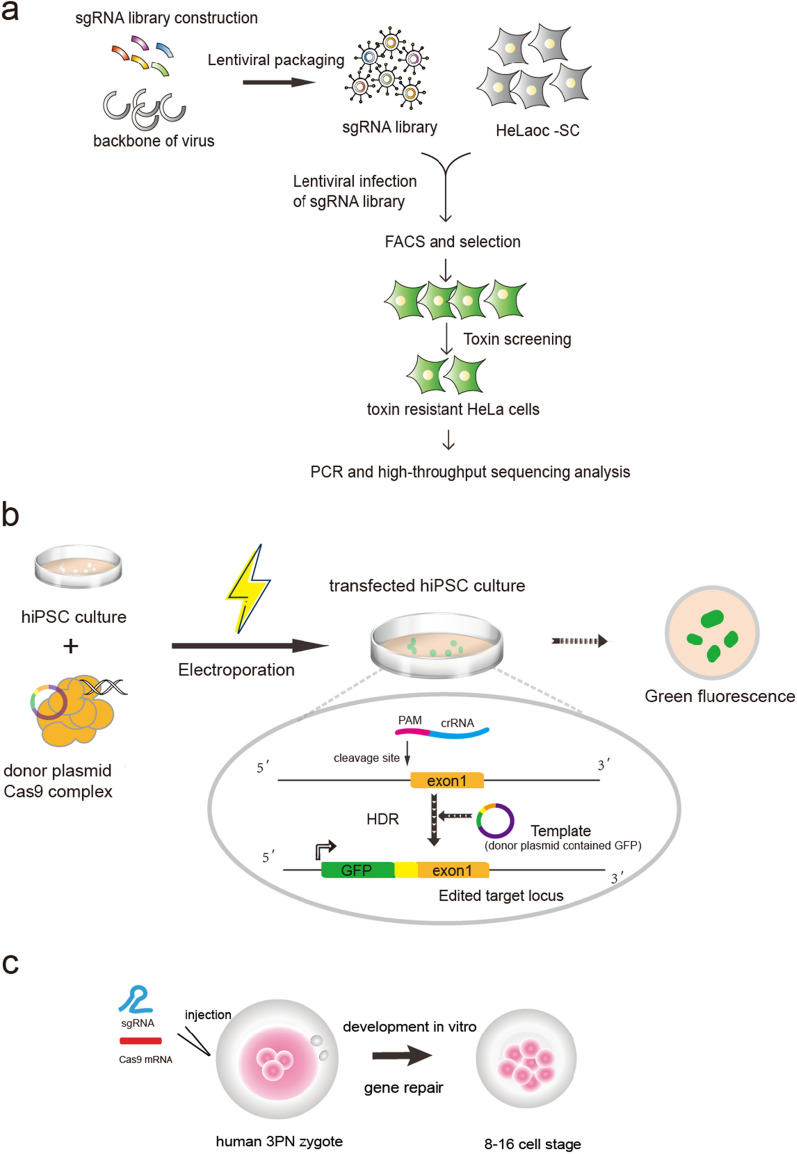
Schematic diagram showing application of CRIPSR/Cas9 system in human cells. **a** Schematic diagram showing the construction of sgRNA library and functional screening steps. These sgRNAs were created to target 291 human genes, and the sgRNA library was designed by the assembly to the backbone of the virus. Through the lentiviral infection, the sgRNAs were delivered into HeLaoc-SC cells and those cells stably expressing sgRNAs were selected by FACS for green fluorescence. After toxin treatment, the resistant cells were selected for PCR and high-throughput sequencing analysis. **b** Schematic depicting the genome-editing process. Human iPSCs were edited using Cas9 complex and donor plasmid by electroporation. Details for genome-editing experiments are shown and the GFP+ cells were collected and used for imaging. **c** Diagram of the injection of Cas9 mRNA and sgRNA into human 3PN embryos to edit target genes, and 8–16 cell stage embryos were further collected to examine genome editing efficiency

Outside of mice and pigs, CRISPR/Cas9 technology is also widely employed in human cells. To conduct large-scale, loss-of-function screens, CRISPR/Cas-based knockout libraries were generated by delivering diverse sgRNAs into HeLaoc-SC cells, and the essential host genes for cell intoxication were identified through anthrax and diphtheria toxin selection (Fig. 4a) [78, 79].

Due to the particularity of human samples, several studies on CRISPR/Cas9 in human stem cells are also meaningful, laying the foundation for subsequent research on oocytes and embryos [80, 81]. Previous investigations have demonstrated that the dCas9-VP64 editing tools targeted by sgRNA can increase the expression of certain genes in human cells [62]. For DNA methylation regulation, DNMT3A could be knocked out by CRISPR/Cas9 in human induced pluripotent stem cells (iPSCs) to determine the impact of DNA methylation in cardiomyocytes [39]. Notably, the methylation of the FMR1 promoter was altered by the dCas9-Tet1/sgRNA editing tool to restore the expression of FMR1 in human iPSCs [68]. In addition, endogenous proteins in human iPSCs were systematically tagged using fluorescent markers through the CRISPR/Cas9 system using a genome-editing strategy (Fig. 4b) [82]. With the development of CRISPR/Cas9, it can be applied to study various diseases. It was reported that iPSCs from DMD patients could be corrected to exhibit them as an adequate model for studying disease-related mechanisms [52]. The application in hematopoietic stem and progenitor cells (HSPCs) has been widely demonstrated, indicating precise induction and repair mechanisms for genome editing in human cell lines [83, 84]. Human iPSCs derived from Wolfram syndrome 1 (WFS1) patients were collected, and disease-causing mutations were successfully corrected using CRISPR/Cas9 technology [85]. Several other diseases, including amyotrophic lateral sclerosis (ALS), sickle cell disease (SCD), and brain tumors, can be treated by the CRISPR/Cas9 system [83, 84, 86–90].

**Fig. 5:**
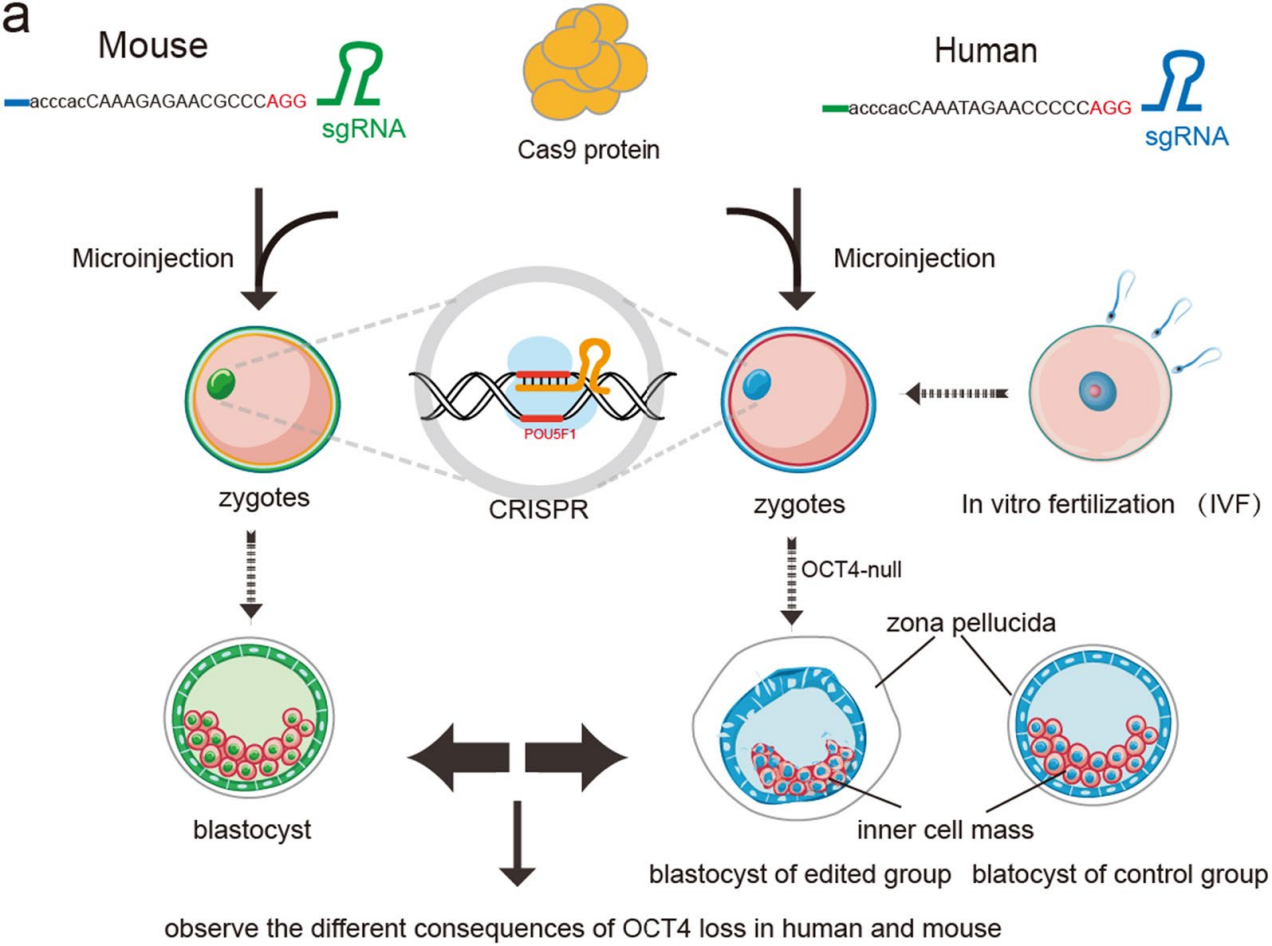
Diagram summarizing the experimental steps of editing POU5F1 (OCT4) locus in mouse zygote (green) and human zygote (blue) using the CRISPR-Cas9 strategy. Different effects on human embryo development were compared in the presence and absence of the OCT4 gene. The role of OCT4 was also compared between mouse and human embryo development

The use of CRISPR/Cas9 to human germline genome editing (HGGE) has been tested in recent years. At first, human tripronuclear (3PN) zygotes which refers to an embryo with three pro-nuclei, were employed for gene editing (Fig. 4c) [91, 92], and later zygotes were used for CRISPR genetic correction of mutations of endogenous β-globin gene (HBB) and glucose-6-phosphate dehydrogenase (G6PD) [93]. With the advancement of microinjection technology, MYBPC3 mutation has been fixed through co-injection of sperm and CRISPR/Cas9 components into human metaphase II (MII) oocytes [35]. By utilizing a zygote microinjection technique, authors designed an efficient sgRNA and targeted the POU5F1 (OCT4) gene with high efficiency (Fig. 5) [34, 35]. They found that loss of OCT4 led to the failure of blastocyst development in humans, suggesting regulatory roles of human OCT4 in cleavage stages [34]. We note that the editing of human mtDNA has also made progress recently [36, 94]. A CRISPR-free editing method called DddA-derived cytosine base editors (DdCBEs), which catalyze C•G-to-T•A conversion through modified DddA deaminase, was reported in 2020 and provided the potential to manipulate mtDNA in human cells [95]. Chen et al. then used the DdCBE technique to edit mtDNA in human 3PN embryos [96]. mtDNA editing has attracted increased attention, especially in oocytes and early embryos, and may become an active research field in the coming years.

Though applying the CRISPR/Cas9 technique in human cells is achievable, the genetic editing results are still unpredictable. In particular, germline gene editing raises a series of social and ethical issues or even laws due to the uncertainty of heritable changes in humans [45]. Dr. Jiankui He, who claimed that he had edited the genome of a human embryo and produced babies successfully, has been strongly condemned and arrested [97]. So far, human genome editing for reproduction has been banned in most countries [46]. The application of CRISPR/Cas9 in HGGE is far from clinical application due to the technical, safety and ethical issues. Improvement of the technique is still ongoing, but its value in evaluating and exploring human early embryogenesis and related pathogenesis should not be underestimated [45].

CRISPR/Cas9 technology has been validated to be feasible in human cells, including early embryos. This technique can be used to explore critical regulatory factors in embryonic development and to facilitate the treatment of human diseases by editing genomic sequences and epigenetic modifications. We note that ethical problems inevitably exist in the operation of human embryos. Subsequent research should further explore current knowledge that troubles the public within the scope of ethics to promote the development of science and the treatment of diseases.

## Challenges and improvements of the CRISPR/CAS9 system

### On-target mutagenesis

With the development of the CRISPR/Cas9 technique, it is gradually found that this gene editing tool might lead to significant mutagenesis [41, 98, 99]. Many articles have shown that applying the CRISPR/Cas9 system caused severe DNA repair errors, which induced genomic damage, including deletion and rearrangement [41, 98, 99]. Both PCR pairing and terminal whole genome sequencing (WGS) revealed large gene deletions in the edited products, and it has been reported that injection of CRISPR/Cas9 components into mouse zygotes even produced a 293 kb gene deletion [41, 99]. Intriguingly, it has been shown that using a single nickase is more effective than the nuclease method in this genome editing system [100]. However, due to the limitations of current detection methods, it may not be possible to assess this large amount of genetic damage accurately [99].

### Off-target effects

Off-target effect is another foremost challenge, and it generates uncertainties including unexpected mutations, deletions, rearrangements, and even cell death during genome editing, which affect the general use of the CRISPR/Cas9 system [101]. To limit the off-target efficiency, engineered Cas9 variants have attracted increasing interest, including mutants of type II *Streptococcus pyogenes* Cas9 (spCas9) with enhanced specificity (eSpCas9) and high-fidelity (SpCas9-HF1) [42, 102–108]. There is also a new hyper-accurate Cas9 variant (HypaCas9) which showed high genome-wide specificity in human cells, and a novel cytosine base editors (CBEs) with rAPOBEC1, which reduced the off-target effects by changing the structure of Cas9 [42, 102–105].

Additionally, it was determined that the activity of the HNH nuclease domain influenced the cleavage of DNA strands. The correct on-target DNA ensures proper cleavage of the double strand by a signal that causes the conformational change of the HNH domain, which is not present when off-target [109]. Subsequently, methods like CHANGE-seq and DISCOVER-seq were reported to be used to inquire about the activity of CRISPR/Cas9 nucleases and the off-target activity [110, 111]. Typically, high-fidelity Cas9 variants reduce the off-target efficiency but the on-target efficiency is also significantly reduced [43]. Unexpectedly, a recent study suggested an intriguing research direction. The Cas9-activated intermediates were investigated and the role of the formation of the guide RNA–DNA target strand duplex and recombination loop in the RuvC domain was found in the mismatch situation. And this mismatch-stabilization mechanism was employed to design SuperFi-Cas9 to reduce the off-target efficiency while maintaining the on-target efficiency [43].

The effect of PAM sequences on the CRISPR/Cas9 system cannot be ignored. A particular Cas9 variant known as xCas9 was identified to be more compatible with different PAM sequences and could reduce off-target effects to improve editing efficiency [108, 112].

### Other effects

After CRISPR/Cas9 takes action, DSB is produced and repaired by HDR, NHEJ or MMEJ [14]. Usually, the efficiency of desired gene repair by HDR is low, and NHEJ which mediated small indels can occur faster and more efficiently, while it usually causes uncontrollable genetic damages [14, 113]. Hence, many methods have been presented to increase the rate of HDR by adjusting the size of the insert/donor, modifying DNA donors with phosphorothioation and inhibiting NHEJ activity [114]. For instance, it was reported that a longer homologous arm and single-stranded oligonucleotide DNA template increased the HDR rate [115]. Inhibiting specific DSB repair pathway regulators like 53BP1 or fusion of Cas9-guide RNA ribonucleoprotein (RNP) complex and a single-stranded oligodeoxynucleotide (ssODN) have been proven to be helpful in improving HDR rate [114, 116]. Moreover, the HDR rate could be increased if CRIPSR/Cas9 is used during HDR-preferred phases (S/G2) in human hematopoietic stem cells [117].

Another challenge using the CRISPR/Cas9 system to edit is high mosaicism, which results in various mutations at the target locus and induces uncontrollable effects on subsequent development [118]. Changing the timing of gene editing has been proven to reduce mosaicism, such as when sperm and CRISPR components are co-injected into MII human oocytes for editing [35]. In addition, the authors indicated that except for the CRISPR/Cas9’s innate separate or enzymatic modification mechanism, the frequent retroelement insertions exacerbate the diversity of alleles and mosaicism in early mouse embryos [44].

## Conclusion and discussion

CRISPR/Cas9 system with simple components has recently been widely applied to mammalian oocytes and embryos, providing great convenience for scientific research and the exploration of disease treatment [34–40]. As an editing technology, the CRISPR system can act on mammalian genomes in cell lines, oocytes, and embryos to correct gene mutations and produce offspring with normal gene expression, which is beneficial for treating related diseases [34–40]. The improved CRISPR technology contains a dCas9 with transcriptional regulators or modifying enzymes, making it possible to regulate the expression of endogenous genes [37, 61–66]. In addition, this technique has been applied in protein visualization, protein-protein interaction and performing large-scale loss/gain of function screening [73, 75, 78]. Even though the CRISPR/Cas9 technology is convenient, there are many limitations and challenges, including on-target mutagenesis, off-target effects and other effects like high mosaicism rate [41–44]. To improve this technology, researchers attempted to design a more efficient editing system by modifying the Cas9 enzyme, studying the nuclease working domain, changing the PAM sequence, improving the HDR rate, and reducing the mosaicism rate [42, 43, 108].

In recent years, CRISPR/Cas9 has been widely used in animal models such as mice, and its application in humans has attracted more attention. Some studies have successfully used this technology to answer scientific questions and tackle disease treatment difficulties [91, 92], but potentially subsequent adverse effects seriously limit its application [41–44]. Editing human embryos involves ethical concerns that make it difficult to advance further, and it may take years to refine the technology and successfully apply it to the editing of the human genome [45, 46]. Particularly, maternal mtDNA is essential for embryonic development. With its excellent targeting performance, CRISPR/Cas9 has successfully edited mtDNA in mice and humans, providing new ideas for studying mitochondrial functions and mitochondria-related diseases [60, 95, 96].

Related mechanisms remain largely unexplored because of the lack of cell models and paucity of studying materials. Subsequent studies of this technique should focus more on limitations and ethical issues, and with the improvement of detection technology, the application will be broader and deeper in mammals. CRISPR/Cas9 system has been regarded excellent genome editing tool and has proven to work well in oocytes and embryos. We believe further improvements will expand its application and minimize its unexpected consequences.

### Supplementary Information

**Additional file 1: Table S1.** Advantages and disadvantages of CRISPR systems.

## Data Availability

Not applicable.
