# Supplementary material for: CRISPR/Cas9 technology: applications in oocytes and early embryos
Source: J Transl Med. 2023 Oct 24;21:746. doi: 10.1186/s12967-023-04610-9 (PMC10594749; doi:10.1186/s12967-023-04610-9)
Supplement: Supplementary file 1 — Additional file 1: Table S1. Advantages and disadvantages of CRISPR systems. [file 12967_2023_4610_MOESM1_ESM.doc]

**Table S1. Advantages and Disadvantages of CRISPR systems**

|  | **Gene editing** by CRISPR/Cas9 technology | **Transcriptional regulation** by CRISPR/Cas9 technology | **Protein targeted regulation and visualization** by CRISPR/Cas9 technology |
| --- | --- | --- | --- |
| **Advantages** | Manipulate DNA sequences directly  Generate knockout animals  Investigate genome variations  Correct genetic mutation diseases  Application is more extensive and variable | Regulate gene transcription activity directly  Manipulate various epigenetic modifications  Investigate transcriptional regulatory networks  Correct certain nongenetic diseases  No disruption of the genome | Add the tags to proteins directly  Fuse degradation/fluorescence tags to proteins  Investigate protein dynamics  Visualize and localize endogenous proteins  No disruption of the genome theoretically |
| **Disadvantages** | Disrupt the genome  Sensitive to unpredictable on/off-target effects  Sensitive to HDR rate | Cannot change DNA sequences  Cannot correct genetic mutation diseases  Sensitive to unpredictable on/off-target effects | Cannot change DNA sequences or gene expression  Cannot correct genetic mutation diseases  Sensitive to unpredictable on/off-target effects |
